# Identifying Principles for the Construction of an Ontology-Based Knowledge Base: A Case Study Approach

**DOI:** 10.2196/medinform.9979

**Published:** 2018-12-21

**Authors:** Xia Jing, Nicholas R Hardiker, Stephen Kay, Yongsheng Gao

**Affiliations:** 1 Department of Social and Public Health College of Health Sciences and Professions Ohio University Athens, OH United States; 2 School of Human and Health Sciences University of Huddersfield Huddersfield United Kingdom; 3 THISC Ltd Manchester United Kingdom; 4 International Health Terminology Standards Development Organization London United Kingdom

**Keywords:** cystic fibrosis, knowledge base, knowledge representation, molecular genetics information, ontology, OntoKBCF, phenotypes

## Abstract

**Background:**

Ontologies are key enabling technologies for the Semantic Web. The Web Ontology Language (OWL) is a semantic markup language for publishing and sharing ontologies.

**Objective:**

The supply of customizable, computable, and formally represented molecular genetics information and health information, via electronic health record (EHR) interfaces, can play a critical role in achieving precision medicine. In this study, we used cystic fibrosis as an example to build an Ontology-based Knowledge Base prototype on Cystic Fibrobis (OntoKBCF) to supply such information via an EHR prototype. In addition, we elaborate on the construction and representation principles, approaches, applications, and representation challenges that we faced in the construction of OntoKBCF. The principles and approaches can be referenced and applied in constructing other ontology-based domain knowledge bases.

**Methods:**

First, we defined the scope of OntoKBCF according to possible clinical information needs about cystic fibrosis on both a molecular level and a clinical phenotype level. We then selected the knowledge sources to be represented in OntoKBCF. We utilized top-to-bottom content analysis and bottom-up construction to build OntoKBCF. Protégé-OWL was used to construct OntoKBCF. The construction principles included (1) to use existing basic terms as much as possible; (2) to use intersection and combination in representations; (3) to represent as many different types of facts as possible; and (4) to provide 2-5 examples for each type. HermiT 1.3.8.413 within Protégé-5.1.0 was used to check the consistency of OntoKBCF.

**Results:**

OntoKBCF was constructed successfully, with the inclusion of 408 classes, 35 properties, and 113 equivalent classes. OntoKBCF includes both atomic concepts (such as amino acid) and complex concepts (such as “adolescent female cystic fibrosis patient”) and their descriptions. We demonstrated that OntoKBCF could make customizable molecular and health information available automatically and usable via an EHR prototype. The main challenges include the provision of a more comprehensive account of different patient groups as well as the representation of uncertain knowledge, ambiguous concepts, and negative statements and more complicated and detailed molecular mechanisms or pathway information about cystic fibrosis.

**Conclusions:**

Although cystic fibrosis is just one example, based on the current structure of OntoKBCF, it should be relatively straightforward to extend the prototype to cover different topics. Moreover, the principles underpinning its development could be reused for building alternative human monogenetic diseases knowledge bases.

## Introduction

“The Semantic Web is a vision for the future of the Web, in which information is given explicit meaning, making it easier for machines to automatically process and integrate information available on the Web” [1]. The major objectives of the Semantic Web are to improve data reuse, data sharing, and data integration in the data-centric Web era [2]. The World Wide Web, in contrast, is document-centric, whereby a document, not the data, is the basic processing unit. Therefore, the Semantic Web, owing to a finer granularity, has the potential to achieve a more precise result in its application.

Ontologies are key enabling technologies for the Semantic Web [3]. Ontologies play a key role in information use via knowledge sharing and reuse [4]. According to the Oxford Living Dictionary, an ontology is defined as “a set of concepts and categories in a subject area or domain that shows their properties and the relations between them.” Another definition by Tom Gruber is “an ontology is a specification of conceptualization” [5]. In this paper, we use ontology to refer a collection of representations of the domain knowledge units (ie, entities and properties) and their relationships that can be utilized by humans, databases, and applications. The Web Ontology Language (OWL) is a semantic markup language for publishing and sharing data by ontologies on the Web [1,6]. OWL is intended to be used in information processing by machines, as well as understood by human beings [1,6]. Description logic can be subjected to automated reasoning to check the consistency of ontologies and determine subsumption relationships [6].

The specific application of ontologies includes standard conceptual vocabularies, services for queries, and reusable knowledge bases, all of which can facilitate interoperability across different systems [7]. Biomedicine is one of the active application domains of ontologies. The *motivation* of our work is to demonstrate the feasibility of providing formal and computable clinical information and molecular genetic information, derived from an ontology-based knowledge base, via an electronic health record (EHR) interface. Molecular genetic information plays a key role in medicine; this information, however, is not the kind that is traditionally included in an EHR system. Therefore, we aim to build an Ontology-based Knowledge Base prototype on Cystic Fibrosis (OntoKBCF) [8,9] to supply formal and machine-processable, clinically relevant molecular genetics information dynamically via an EHR prototype.

Cystic fibrosis is used as an example disease in building OntoKBCF for a feasibility demonstration. The reasons for this choice include the following: cystic fibrosis has a relatively stable and comprehensive molecular genetic profile; it is one of the most common, fatal genetic diseases in the United States [10], especially in white people of Northern European ancestry [11,12]; the mechanism of the disease has been well studied and well understood; and cystic fibrosis is a single gene disease, which is less complicated than multiple-gene diseases. The cystic fibrosis transmembrane conductance regulator (*CFTR*) gene causes cystic fibrosis.

An ontology-based knowledge base is built on top of domain entities, properties, and relationships (ie, the ontology); however, it also includes sharable instances, rules, and inference capabilities [13], which extend the ontology. Therefore, an ontology-based knowledge base can be utilized via an EHR system, for example, by incorporating the characteristics of patients. In this paper, we use a *knowledge base model* to refer to the logic specifications of a knowledge base at the abstract level and a *knowledge base prototype* (ie, OntoKBCF) to refer to the physical artifact that we eventually built at the concrete level, based on a knowledge base model. We use a *knowledge base prototype* instead of a *knowledge base* because we include different levels of facts in OntoKBCF, for example, nucleotide changes, amino acid changes, and clinical phenotypes. The facts are not exhaustive at any horizontal level within OntoKBCF; however, the facts are sufficient to organize the relationships at cross-horizontal levels [8,9] and to demonstrate how to generate a knowledge resource including molecular genetics information and health information via an EHR prototype [14,15].

An overview of the project, showing how OntoKBCF supplies computable and formally represented molecular and health information to an EHR in a dynamic manner has been published previously [14], as has the technical integration of OntoKBCF and the EHR prototype [15]. A further related source [8], published at an early stage of the project, mainly describes the *domain coverage* of OntoKBCF. In contrast, this paper provides *a holistic view of the construction principles* of OntoKBCF, including the *representations of the integration* between OntoKBCF and an EHR. As such, this paper may provide a reference for content developers operating in the Semantic Web space, especially in building knowledge bases and knowledge base modeling on monogenetic human diseases.

## Methods

### Representation Language and Terms Used in Ontology-Based Knowledge Base Prototype on Cystic Fibrosis

**Figure 1:**
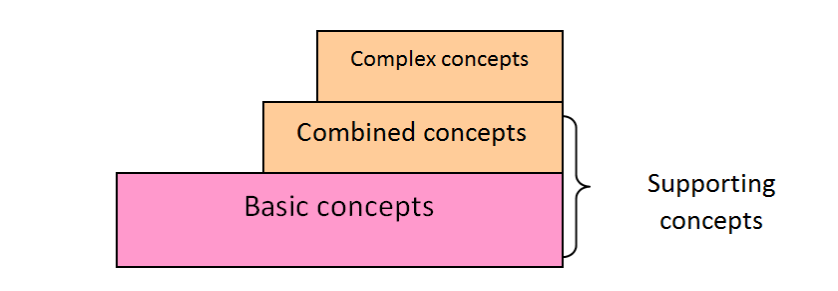
Relationships between construction terms used in this paper and their relative sizes.

OntoKBCF was built using Protégé-OWL [16]. We chose OWL description logic (DL) [1] as the language for OntoKBCF. We used basic, combined, and complex concepts, as well as final facts, to represent selected content within OntoKBCF. Table 1 provides definitions for the major terms that we have used in the remainder of the paper, with examples. Figure 1 shows the relationships between the terms and their relative volumes of each category within OntoKBCF.

**Table 1:** Major construction terms used in the paper.

Term name	Annotation and example
Basic concept	The atomic concept from Unified Medical Language System [17], Gene Ontology [18], or domain knowledge, such as Gly, which is the amino acid glycine.
Combined concept	Combination of two or three basic concepts, such as Gly542, which is Gly’s location, 542 in the cystic fibrosis transmembrane conductance regulator amino acid chain.
Complex concept	Combinations of more than three basic concepts and usually they are subjects. A subset of the combined concepts is explained through the EHR^a^ interface, such as Patient_CF_with_Gly542X, which is a group of cystic fibrosis patients with the Gly542X variant.
Supporting concept	Basic concepts and combined concepts, used in explaining the complex concept.
Final fact	A domain statement represented in OntoKBCF^b^ through a combination of basic concepts or combined concepts with properties and logic relationships, such as property and description of Patient_CF_with_Gly542X.
Fact	Any concept above is a subset of facts, which includes (1) hierarchy of concepts, both basic and combined concepts, and (2) a property description of the concept.

^a^EHR: electronic health record.

^b^OntoKBCF: Ontology-based Knowledge Base prototype on Cystic Fibrosis.

### Organization and Main Construction Procedures of Ontology-Based Knowledge Base Prototype on Cystic Fibrosis

**Figure 2:**
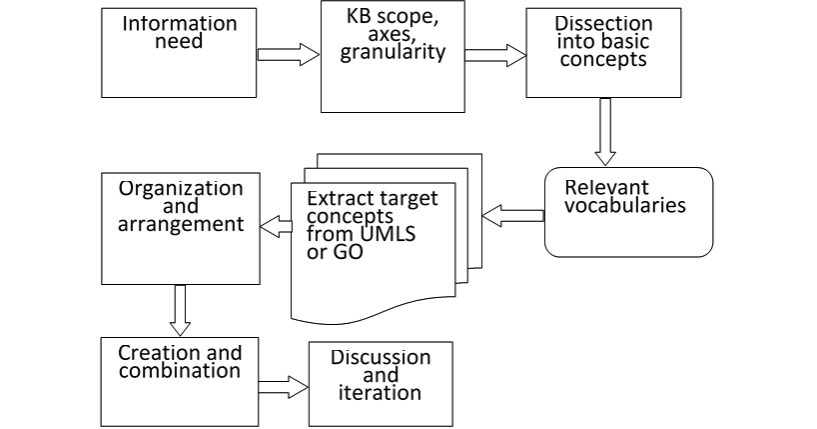
General construction procedures of the ontology-based knowledge base prototype on cystic fibrosis. KB: knowledge base; UMLS: Unified Medical Language System; GO: gene ontology.

The main axes used in OntoKBCF are time and problem in line with the content of the included domain. The main axes are utilized to organize the atomic, combined, and complex concepts in OntoKBCF. Figure 2 shows the main construction processes for OntoKBCF.

### Scope of Ontology-Based Knowledge Base Prototype on Cystic Fibrosis and Sources

**Figure 3:**
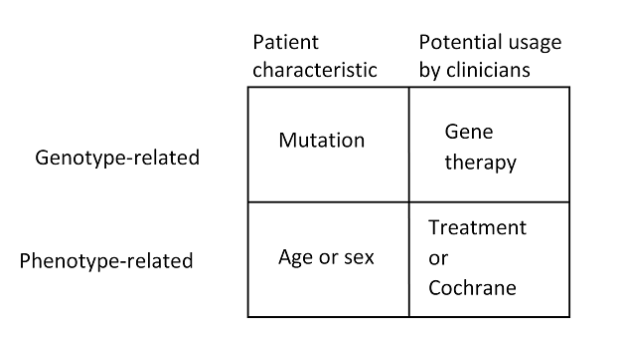
Main content categories represented in the ontology-based knowledge base prototype on cystic fibrosis and their potential usage.

Figure 3 presents the main content of OntoKBCF. One of the purposes of OntoKBCF is for clinical use; therefore, the following factors were considered when determining domain content: (1) treatment or therapy, which has been reported as the main category of information needs for clinicians, including gene therapy and other treatments for cystic fibrosis [19-25]; (2) availability of relevant molecular genetic information, such as the most common *CFTR* mutations, particularly those related to health information or phenotypes (eg, symptoms, ethnic groups) [26-28]; (3) cystic fibrosis has a time-related development cycle, and usually, a patient’s age is available in an EHR; thus, time-oriented descriptions of cystic fibrosis are included in OntoKBCF [21,23-25].

### Top-to-Bottom Content Analysis in Ontology-Based Knowledge Base Prototype on Cystic Fibrosis

The selected content required analysis to set up the construction strategies. Based on the scope and the knowledge resources available, analyzing the facts by utilizing a series of dissections was necessary to make the task realistic and practically accomplishable. The starting point was sentences of facts that were dissected into atomic units (ie, basic concepts in Figure 1). The atomic units were represented with their necessary hierarchies, they were combined, and their properties included to express the complex facts in a logical, machine-processable format. Usually, complex facts can be represented by one or multiple concepts, described in Figure 1, and their properties.

**Figure 4:**
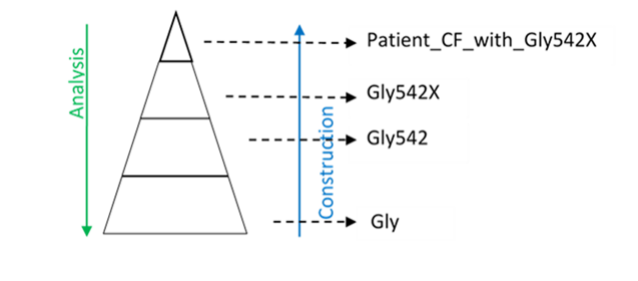
General analysis (top-to-bottom) and construction (bottom-up) for ontology-based knowledge base prototype on cystic fibrosis.

The general criteria of the dissection were determined by the scope and granularity of OntoKBCF; otherwise, dissection would be an endless process. Only necessary superclass concepts, rather than detailed and complete subsets of Unified Medical Language System (UMLS) [17] and Gene Ontology (GO) [18], have been included in OntoKBCF. Only the properties and constraints within the scope of OntoKBCF, rather than a complete and comprehensive description for each class, are represented. The main reason for doing so is to ensure that the tasks are accomplishable with limited resources and within a restricted timeline. The dissection process was followed by reconstruction. Figure 4 shows the analysis and reconstruction order that we utilized in constructing OntoKBCF.

### Bottom-Up Construction Examples in Ontology-Based Knowledge Base Prototype on Cystic Fibrosis

OntoKBCF was constructed bottom-up [29] as follows. After dissection analysis, reconstruction work started from basic atomic concepts. Then, the basic concepts were modified step-by-step until the basic concept was turned into a meaningful composite (a combined or complex concept). Every later concept used the former ones in its representation. Then, final facts were represented by the combination of a subject (ie, its definition) and its properties. A combination of basic concepts was utilized to build combined and complex concepts. The complex concept could be utilized as the subject to describe characteristics for a subgroup of patients. Figure 4 shows the construction process of OntoKBCF.

By way of example, if a group of patients with the Gly542X variant needs to be represented, the following steps would be used (see Figure 4):

- “Gly” stands for glycine, a type of amino acid; this is a basic concept.
- “X” is the nonsense codon, which terminates the translation; this is a basic concept.
- “Gly542” is defined as an amino acid location in the human *CFTR* amino acid chain; this is a combined concept.
- “Gly542X” is defined as an amino acid substitution in human *CFTR* amino acid chain; this is a combined concept.
- “Patient_CF_with_Gly542X” is defined as a group of cystic fibrosis patients with the amino acid change; this is a complex concept.
- Final facts about the group of cystic fibrosis patients with the Gly542X variant are represented by the combination of both the logical definition of “Patient_CF_with_Gly542X” and its properties.

Most of the hierarchies of basic concepts in the knowledge base prototype follow UMLS and GO. In addition, relationships or classes were added or adjusted if there were no obvious choices. For example, in OntoKBCF, “sex_group” was used to connect “population_group” and “female,” and the concept “*CFTR* gene” in UMLS was adapted to “Human *CFTR* gene”; considering the clinical usage potential, we distinguished the human origins of the *CFTR* gene.

### Intersection and Combination Representation Principles

**Figure 5:**
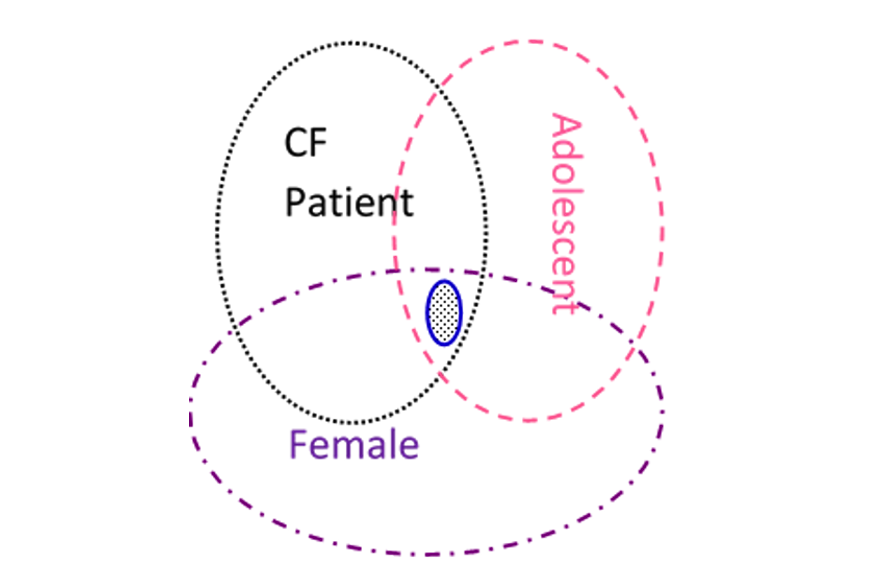
Representation of adolescent female cystic fibrosis (CF) patient in ontology-based knowledge base prototype on cystic fibrosis.

**Figure 6:**
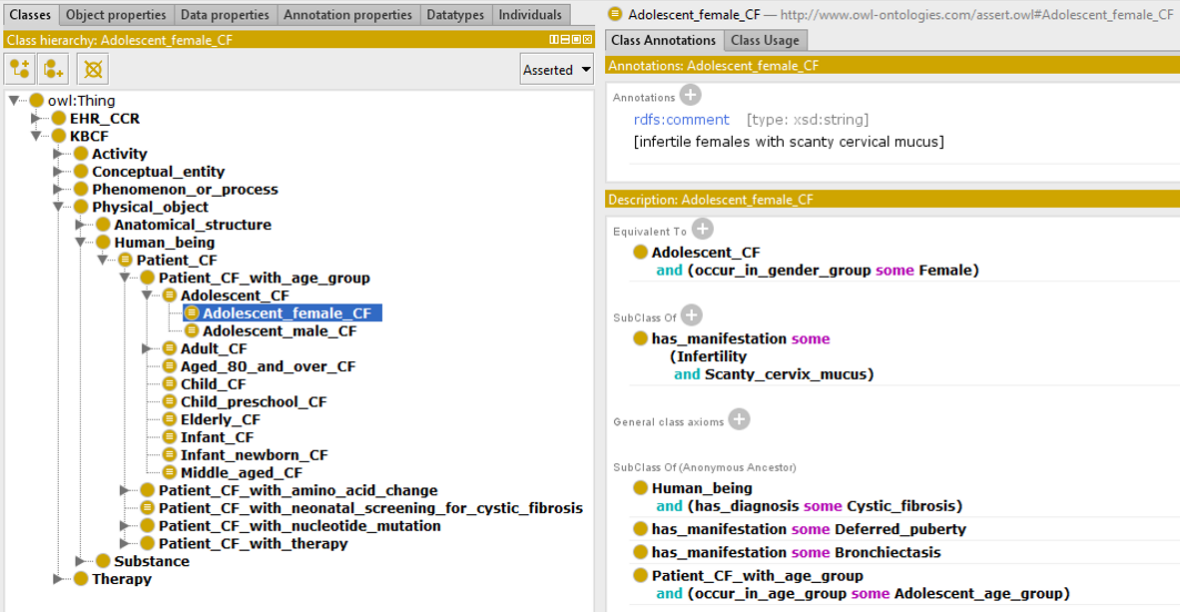
The representation of an adolescent female cystic fibrosis patient group in ontology-based knowledge base prototype on cystic fibrosis via Protégé-Web Ontology Language. (Protégé Source: Standford University).

We used the intersection among basic concepts to represent combined and complex concepts in OntoKBCF. For example, “female adolescent cystic fibrosis patient” is represented as a complex concept in OntoKBCF. Figure 5 shows the intersectional representation principle in OntoKBCF for female (ie, the purple circle), adolescent (ie, the rose circle), and cystic fibrosis patients (ie, the black circle) as a group, which represents *all* possible properties of this group. Figure 6 shows the exact representation (ie, final facts are represented as the combination of a definition of a complex concept and all its properties on the right lower corner of the figure) that we created within Protégé-OWL. The description in Figure 6 is a subset of the intersection in Figure 5. The entire set of properties for the subject (intersection of 3 bigger ellipses), compared with the filled ellipse (in blue) represented in OntoKBCF, refers to all the possible properties related to an adolescent (in rose) female (in purple) CF patient (in black). The reasons that we did not represent all possible properties are listed in the section Top-to-Bottom Content Analysis in OntoKBCF.

### Ontology-Based Knowledge Base Prototype on Cystic Fibrosis Construction Principles

Our *philosophy* is to use the existing concepts as much as possible and to create new ones only if it is absolutely necessary. If a concept is from UMLS, then the unique concept identifier from UMLS is kept in the Annotations section for future reference (eg, reuse, mapping, and communication). Other construction *principles* include the following: (1) to represent more types (eg, nucleotide deletion, insertion, transition, and transversion) of facts within the same level; (2) to represent 2-5 examples within the same type, for example, for the single-nucleotide transition of *CFTR*, we represented 5 examples in OntoKBCF instead of representing all existing facts exhaustively; (3) to have the same representation listed in all possible hierarchies in OntoKBCF; for example, *AA2183 _minus_G* is listed under *Del_A*, *Nucleotide_deletion_in_ human_CFTR_gene*, *A_transition_G*, and *Nucleotide_ transition_in_human_CFTR_gene*; and (4) to represent only the facts within the scope of OntoKBCF. The purpose of our work is to demonstrate the feasibility of the construction of a knowledge base by utilizing Semantic Web technology and demonstrating that the facts from the knowledge base can be utilized via an EHR interface dynamically. Therefore, representing the exhaustive list of variations of the same gene is not a priority. In addition, as most of the mutation types have been covered in the current OntoKBCF, we believe it to be extendable. To include an exhaustive list of the variations of the *CFTR* gene should be straightforward when following our construction principles and approaches.

### Naming Conventions in Ontology-Based Knowledge Base Prototype on Cystic Fibrosis

To create the potential for broader usage, some of the classes were specifically named for human beings in OntoKBCF, such as “Human_CFTR_gene” and “Human_CFTR_gene_exon”; this differentiates them from any additional classes that may concern other organisms.

The Nomenclature for the Description of Sequence Variations [30] is followed in OntoKBCF. In addition, the following basic name rules are used in OntoKBCF: (1) for amino acid changes (ie, protein level), a 3-letter abbreviation name was used; (2) for coding DNA (cDNA)-level nucleotide changes, a 1-letter name (such as “A,” “T,” “C,” and “G”) was used. These design decisions are present in OntoKBCF as follows: (1) a mutation name, which strictly follows the nomenclature recommendation [30,31], has been kept in the “Annotation” section and (2) there were only cDNA- and protein-level descriptions of the *CFTR* mutation name, which did not include the RNA-level description. The reason is that cDNA is used to code protein, and RNA is more of a middle layer with regards to protein synthesis. Amino acids can be named in 1 or 3 letters. There are both amino acids and nucleotides in OntoKBCF, and 1-letter abbreviation names of amino acids can be confused with nucleotides. For example, A can be Alanine (an amino acid; the 3-letter abbreviation is Ala) or Adenine. Thus, we use 3-letter abbreviation names for amino acids and 1-letter names for nucleotides.

As there are 22 types of amino acids, there are many more possibilities for amino acid changes compared with nucleotide mutations; only the amino acid changes that fell within the scope of OntoKBCF were included. In contrast, because there are 8 types of nucleotides, which consist of DNA and RNA, with a smaller number of possible mutations, the complete set of possible nucleotide mutations in cDNA for deletion, insertion, transversion, and transition were included in OntoKBCF. The symbols “+” or “−” indicate the nucleotide position at the beginning of the intron (+) or the end of the intron (−); this would be represented as “plus” and “minus” in OntoKBCF for nucleotide substitutions. Amino acid substitutions are handled similarly.

In OntoKBCF, the final expression of nucleotide mutations can be distinguished from amino acid changes as follows: (1) their labels contained “minus” or “plus,” or “Ins” or “Del” (such as “AA2183_minus” or “G621_plus_1”, and “Del394” or “Ins3905”); or (2) a 3-letter abbreviation name was used for amino acids to avoid confusion. For example, Gly was utilized as the abbreviation for glycine, not G, which can be confused with the nucleotide “G.” “Minus” and “plus” were used to represent the nucleotide position. “Ins” is used for nucleotide insertion, and “Del” is used for nucleotide deletion. In theory, not all nucleotide mutations have to be labeled with one of these 4 values; however, no such example has been found in the construction of OntoKBCF. This strategy avoids the potential limitations of the special characters that one can use in a name.

### Other Considerations in Ontology-Based Knowledge Base Prototype on Cystic Fibrosis

For properties in the OWL representation, only existential restriction “someValuesFrom” was used as the property restriction (appearing as “some” in the text); it would be inappropriate to use allValuesFrom (appearing as “only” in the text,) because it cannot be guaranteed that their value classes are fully specified and mutually exclusive and exhaustive. It is sufficient to use existential restriction in most representations to leave space for future efforts to complete the description. For example, for a cystic fibrosis patient with Gly542X, the subject had the mutation property “some Gly542X” because it is possible that Gly542X is not the only mutation for this type of patient.

According to Horridge et al [32], the “Domain” and “Range” conditions in the “Property” tab of the Protégé-OWL interface are not used as a constraint. These properties are outside of the scope of this project, and consequently, the “Domain” and “Range” widgets were left blank for all properties.

## Results

### Ontology-Based Knowledge Base Prototype on Cystic Fibrosis Content Summary

Table 2 summarizes the metrics of OntoKBCF. Description logic expressivity was ALCHIF (attribute language; complex concept negation; role hierarchy; inverse properties; functional properties).

In OntoKBCF, the most fine-grain level of biological information starts at nucleobase, which is the most important component for the elementary units (nucleotides) of RNA and DNA. In contrast, for health information in OntoKBCF, the most fine-grain level starts from relatively *atomic concepts*, such as diarrhea, nausea, or coughing.

Currently, OntoKBCF includes answers to only questions about “what” (eg, what is Gly542X? What is the hierarchy of cystic fibrosis?), not about “how” (eg, disease mechanisms and disease pathways) or “why” (eg, reasons and explanations) [8]. This is a pragmatic approach to make this study manageable and feasible. We focus on “what” questions initially. We do recognize the importance of “how” and “why” questions in clinical usage, especially in explanation of pathophysiological mechanisms and disease pathways. However, we leave these questions for future development. Textbox 1 lists the main categories of content in OntoKBCF. In the Multimedia Appendix 1, we also include a screenshot of all the object properties in OntoKBCF.

OntoKBCF has been shared via BioPortal [9]. We used a reasoner, HermiT 1.3.8.413 within Protégé-5.1.0, to check the consistency of OntoKBCF.

### Ontology-Based Knowledge Base Prototype on Cystic Fibrosis Application Functions in an Electronic Health Record

OntoKBCF has been demonstrated to be able to supply personalized, nonduplicate molecular genetic information and health information according to a patient’s characteristics successfully, dynamically, and automatically via an EHR prototype [14,15]. The connection between OntoKBCF and the EHR prototype can be made through a combination of automatic and manual means. OntoKBCF includes a section for the EHR main structure, which is utilized to map the domain knowledge in OntoKBCF to the EHR structure and to make the connection automatic.

**Figure 7:**
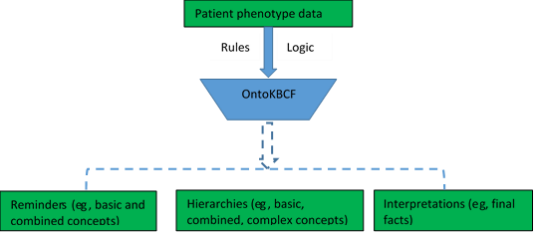
The conceptual architecture between ontology-based knowledge base prototype on cystic fibrosis (OntoKBCF) and an electronic health record interface.

OntoKBCF can provide reference facts (eg, hierarchies of basic concepts, combined concepts, and final facts) and reminders to EHR users. When a user applies the final facts via EHR interfaces, all the basic concepts and combined concepts utilized for building a complex concept can be accessed. Figure 7 shows a conceptual architecture about how OntoKBCF can be utilized within an EHR user interface.

**Table 2:** Metrics of ontology-based knowledge base prototype on cystic fibrosis content summary [9].

Name	Count (n)
Axiom	1375
Logical axiom	665
Declaration axiom	445
Class	408
Individual	0
Object property	35
SubClassOf	498
EquivalentClasses	113
DisjointClasses	37
Maximum depth	9
Maximum number of children	20
Average number of children	3
Classes with a single child	69
AnnotationAssertion	265

**Main categories of content in ontology-based knowledge base prototype on cystic fibrosis.**

**Biological information (genotype-related)**

- Nucleotide mutation
- Amino acid change
- Gene mutation location
- Gene or protein mutation dissection

**Health information (phenotype-related)**

- Demographic data (eg, age, sex, and ethnicity)
- Symptoms
- Diagnostic tests or diagnosis
- Treatment facts

**Electronic health record data representation structure**

## Discussion

### Significance of Ontology-Based Knowledge Base Prototype on Cystic Fibrosis

OntoKBCF meets our design objectives and has been integrated successfully with an EHR prototype [15]. This paper illustrates the construction principles, representation approaches, and challenges in constructing OntoKBCF in Protégé-OWL. We believe that the methods we reported to construct OntoKBCF will provide a useful reference for Semantic Web content developers, particularly in constructing knowledge bases on human monogenetic diseases for clinical usage. The basic and combined concepts and similar facts in both biological and health fields represented in OntoKBCF can be found in other diseases. OWL statements make OntoKBCF sharable and computer processable. The possibility of automated reasoning assures the consistency of the underlying ontology. The organization, structure, and use of existing, controlled vocabularies and terminologies as much as possible within OntoKBCF improve its potential for compatibility and reusability.

### Patient Group Representation Challenge Example

**Figure 8:**
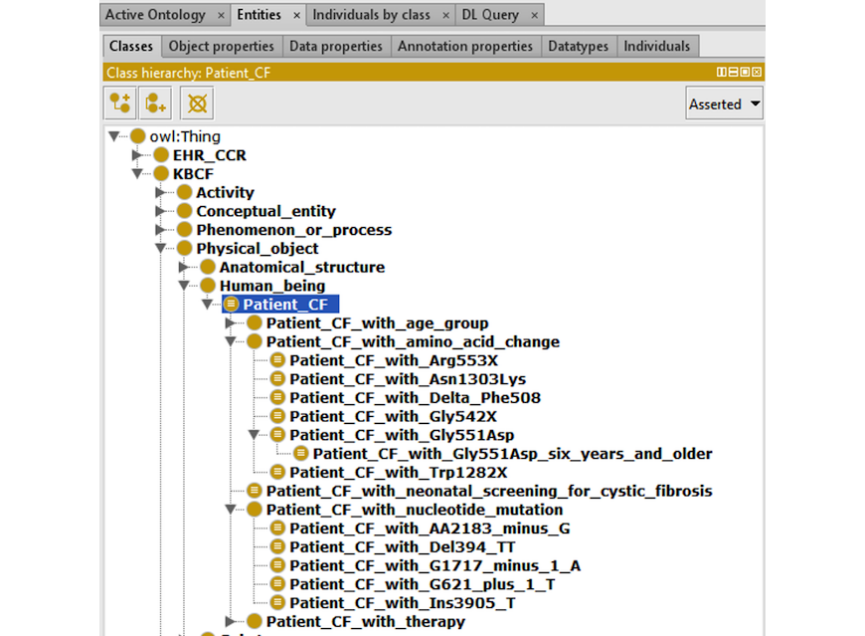
Main cystic fibrosis patient groups in ontology-based knowledge base prototype on cystic fibrosis via Protégé-Web Ontology Language. (Protégé Source: Standford University).

In OntoKBCF, different groups of cystic fibrosis patients are represented separately. For example, there are cystic fibrosis patients in different age groups or with different genetic variations. Figure 8 shows the main categories of cystic fibrosis patient groups included in OntoKBCF.

**Figure 9:**
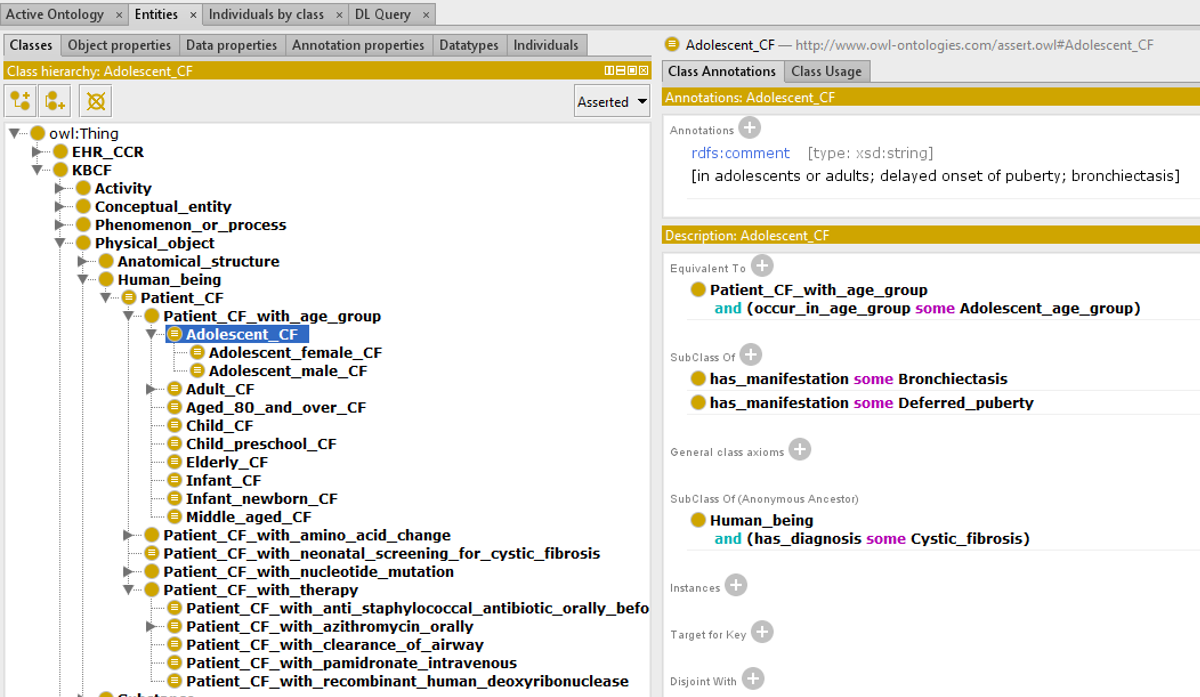
The representation of an adolescent cystic fibrosis patient group in ontology-based knowledge base prototype on cystic fibrosis via Protégé-Web Ontology Language. (Protégé Source: Standford University).

Currently, for each cystic fibrosis patient group, we represent only the available facts from the selected resources and within the scope of OntoKBCF. We defined patient groups logically, rather than representing them comprehensively. Some cystic fibrosis patient groups, such as an adolescent cystic fibrosis patient group (Figure 9), are fairly straightforward, while some are fairly complicated because of available facts. Figure 6 demonstrates a partial representation of the adolescent female cystic fibrosis patient group.

For the complicated groups in OntoKBCF, the representation is far from comprehensive or exhaustive. We feel that if we were to set comprehensive representation as a goal, the task would be endless, as there are many properties related to the patient groups. The properties can include, among other things, clinical manifestations, treatment plans, nursing care plans, patient self-care plans and patient education. The detailed categories, as well as the actual representation for each property, would result in an exhaustive list. Our pragmatic solution to this scalability problem is to focus on practical applications, stay within the defined scope, and make it possible to expand in the future.

### Ambiguous Concepts and Uncertain and Negative Statement Representation Challenge

There are some common expressions in clinical medicine, for example, “sometimes,” “especially,” “very common,” and “very rarely” that are challenging to represent in a computable and formal manner as their interrelationships are vague. These expressions are very important for human experts (such as physicians) to absorb or to apply the information. For example, for older children with cystic fibrosis, one important symptom is “nasal polys, especially if recurrent” [21]. However, we cannot represent this type of fact in a computable form. Likewise, Tao et al [33] recognized that representing uncertain expressions is a challenge in creating a temporal-related ontology.

In addition to uncertain concepts, negative descriptions cannot be represented precisely, either. Considering the limited reasoning support, less than ideal reasoning efficiency, and the complexity in modeling concepts, currently, for demonstration purposes, we included only the definite and positive results in OntoKBCF. We do realize that although this is necessary, it is a limitation of OntoKBCF as it does not reflect the real world comprehensively.

**Figure 10:**
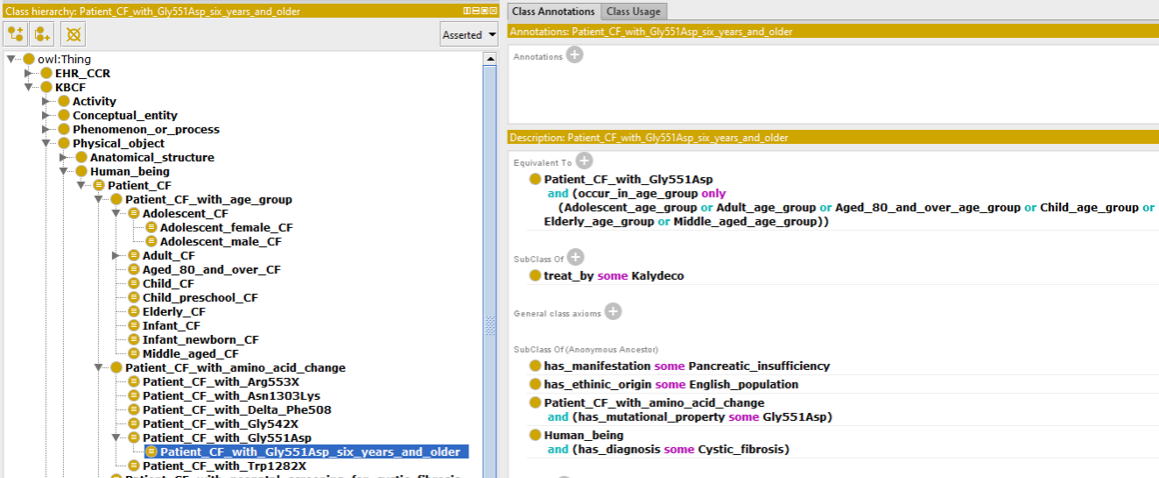
A cystic fibrosis patient group with the Gly551Asp variation and aged at least 6 years represented in ontology-based knowledge base prototype on cystic fibrosis via Protégé-Web Ontology Language. (Protégé Source: Standford University).

### Complex Facts Representation Challenges

One of the complex facts represented in OntoKBCF is shown in Figure 10. This is a representation of a cystic fibrosis patient group whose patients have a mutation from Gly to Asp at 552 positions and are at least 6 years old. The current properties include diagnosis, treatment (ie, medication) information, age groups, ethnic origin, clinical manifestation, and exact amino acid change. This example has positive and definite statements, which are represented in the current OntoKBCF. However, how to represent more complicated facts, such as detailed molecular mechanisms or pathway information, with higher than first-order logic and how to process those descriptions also would be challenging.

### Broader Challenges in the Construction of Ontology-Based Knowledge Base Prototype on Cystic Fibrosis

The challenges of introducing information technologies and computer science applications to medicine have been recognized for a long time [34]. Knowledge representation in medicine is one of the many areas that face issues. It is difficult to say that the challenge is in knowledge representation, *per se*, or that the challenges are in the broader area of medicine, logic, or even mathematics.

Regarding OntoKBCF, we sought to determine how many properties are sufficient or ideal to describe a cystic fibrosis patient group. From a pragmatic point of view, setting up the scope of the project is necessary to make the project manageable. Otherwise, the representation would become an endless task, as there are many possible properties related to a group of cystic fibrosis patients, as Davis et al [35] described. If we assume that we constructed OntoKBCF and others constructed similar knowledge bases about cystic fibrosis, then other management-related challenges would emerge, for example, (1) how to validate classes from different sources; (2) how to match the same classes from different sources; (3) how to integrate the properties for the same class that originates from different sources; (4) how to integrate different ontologies about the same topic; and (5) how to authorize and achieve consensus for the combined classes or ontologies conveniently. Currently, BioPortal [36] is an effective way to collect submitted biomedical ontologies; it can be utilized to check existing ontologies before building any new ontologies. Ontobee [37] provides a finer search on ontology terms, which goes further than index-based searches on ontologies, by providing an accessible unique resource identifier (URI) for ontology terms. These examples provide great potential in reusing and mapping ontology terms; however, many of the abovementioned challenges are still not solved. In addition to using URI, including the coded concepts, such as unique identifiers in UMLS or the clinical terminology systematized nomenclature of medicine-clinical terms, may improve the reusability of the knowledge base in general.

One critical challenge in building OntoKBCF is a lack of sufficient clinical actionable knowledge that is related to molecular genetic information. This is one of the main reasons that we constructed OntoKBCF as a prototype rather than as a product knowledge base.

Other challenges that we faced during the construction of OntoKBCF included the following: (1) how to communicate the construction principles and design considerations in a consistent, explicit, and sharable manner; (2) whether we can make the construction process automatic; and (3) how to update the domain content automatically. Solving these challenges may help us build larger-scale ontologies and help in their integration automatically.

### Other Related Work

Our work was originally conceived in 2004 and mostly completed by 2009. At the time, there were no available resources that could meet *all* of the following criteria: consistent molecular genetic information and a clinical actionable, machine-processable format that was shareable, reusable, and customizable. We, thus, had to create our own resource to meet all the requirements. Biobanks might be a good source to provide such information. Nevertheless, the UK Biobank [38] started to recruit participants only in 2006, and the USA Biobank, as a part of the Precision Medicine Initiative, was founded only in 2016 [39].

We used an ontology-based knowledge base to provide customizable molecular genetics and health information to EHR settings successfully. The ontology-based knowledge base provides potential in reusing and sharing, as well as the consistency of information. The creation of knowledge resources with fine granularity (not only disease names) has been recognized repeatedly [40,41] as the main challenge in bringing new information (eg, molecular genetics information) to an EHR. Although there are many existing databases for both genotypes and phenotypes of human beings, not all of them are organized in a machine-processable format. Therefore, the detailed construction principles and approaches for OntoKBCF reported in this paper can provide a reference to peers in the field.

One recent example of knowledge base was reported by Samwald et al [42,43] who built a resource description framework or OWL knowledge base to provide support for clinical pharmacogenetics. Their work shares some similarities with OntoKBCF. For example, it provides consistent genomics information in clinical settings in a machine-processable format. Both projects use reasoners to maintain consistency. The two projects, however, have different focused application domain areas; Samwald et al’s work focuses on pharmacogenomics information, and OntoKBCF uses cystic fibrosis to demonstrate possible broader applications. In addition, Samwald et al’s work includes a query and answer part that supersedes OntoKBCF. Meanwhile, OntoKBCF demonstrates its usage via an EHR prototype; such demonstration was not included in Samwald et al’s [42,43] publications. There is no detailed description of the knowledge base construction in Samwald et al’s project. Thus, it is difficult to compare the representation and construction principles and approaches of the two projects in depth.

In recent years, the broad importance of Semantic Web technologies has been recognized. There are many more studies that have used ontology-based knowledge bases to assist clinical tasks within an EHR. For example, Robles-Bykbaev et al [40] reported the use of a formal knowledge base model to assist in the generation of decision making and recommendations for communication disorders. They stated that their knowledge base is used to support data analysis and inference processes; however, the paper does not include the details of the organization and structure of the knowledge base. The Clinical Narrative Temporal Relation Ontology (CNTRO) [33,44] is a temporal-related ontology in the OWL. CNTRO is used for inference purposes in processing clinical narratives. The use cases [45] show promising results in processing short and simple adverse event narratives. The OWL query application [46] for CNTRO and the harmonization of CNTRO with other existing time ontologies may improve CNTRO [44] further and provide broader applications in analyzing clinical narratives. Another example is the research of Wang et al [41] and Hu et al [47]. They utilized an ontology-based clinical pathways knowledge base to generate personalized clinical pathways for clinicians by incorporating patients’ data. The clinical pathways knowledge base is independent of an EHR, and it can be shared by other systems. All these examples used ontology-based knowledge bases and integrated them with an EHR. There are other ontology examples that are utilized in settings other than in EHR, such as in mobile devices [48] or to facilitate social media data mining [49] for health purposes. While these recent studies demonstrate the general application of semantic technologies in health care, they do not consider the important clinical usage of relevant molecular genetic information.

### Limitations of Ontology-Based Knowledge Base Prototype on Cystic Fibrosis

OntoKBCF is a knowledge base prototype rather than a knowledge base, which may include substantial instances for each class. OntoKBCF, however, includes content from nucleotides to clinical phenotypes across different granularity levels. If we can image a pyramid that moves from gene to protein to cell to tissue to organ up to human beings [50], we can see that the current OntoKBCF is not thorough at any horizontal level; however, it includes all vertical levels of knowledge. The expansion of each level should not be too challenging, as each horizontal level has some classes represented in OntoKBCF.

There has been no formal evaluation of OntoKBCF. However, we do have two limited levels of validation of the work: (1) automated reasoning assures the consistency of underlying ontology and (2) successful demonstration that OntoKBCF can deliver customizable molecular information and health information dynamically via an EHR prototype. As a knowledge base model, these levels of validation provide evidence that the original design objectives of OntoKBCF were met successfully.

### Conclusion

We have presented the robust construction principles, approaches, design decisions, and challenges in constructing OntoKBCF. OntoKBCF is constructed in OWL and can be utilized as a knowledge resource to provide information on molecular genetics and other health topics dynamically to an EHR. The construction principles and approaches can be referenced for other topic areas, and based on its current structure, OntoKBCF could be expanded in a straightforward manner to a full knowledge base.

## Appendix

Multimedia Appendix 1 Additional screenshot of properties of OntoKBCF.

## Abbreviations

*CFTR*: cystic fibrosis transmembrane conductance regulator
CNTRO: Clinical Narrative Temporal Relation Ontology
EHR: electronic health record
GO: Gene Ontology
OntoKBCF: Ontology-based Knowledge Base prototype on Cystic Fibrosis
OWL: Web Ontology Language
UMLS: Unified Medical Language System
URI: unique resource identifier
