# Supplementary figures and images for: Identifying Principles for the Construction of an Ontology-Based Knowledge Base: A Case Study Approach
Source: JMIR Med Inform. 2018 Dec 21;6(4):e52. doi: 10.2196/medinform.9979 (PMC6320437; doi:10.2196/medinform.9979)

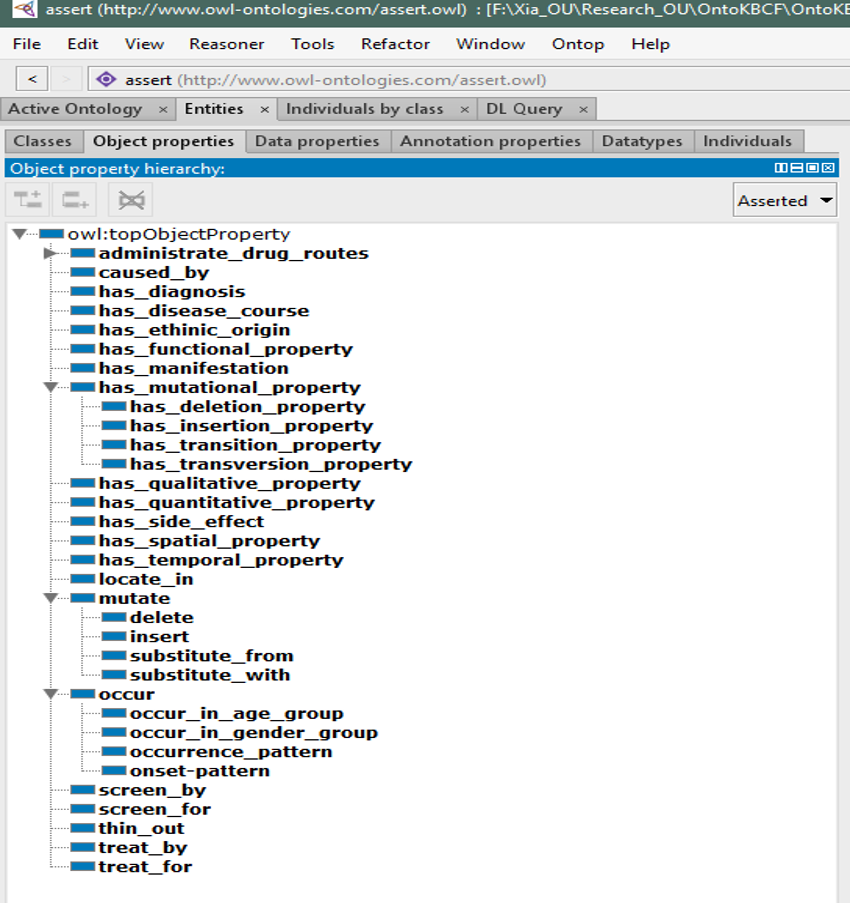

Supplement: Multimedia Appendix 1 [file medinform_v6i4e52_app1.png]
